# *Stevia* Polyphenols, Their Antimicrobial and Anti-Inflammatory Properties, and Inhibitory Effect on Digestive Enzymes

**DOI:** 10.3390/molecules28227572

**Published:** 2023-11-13

**Authors:** Khaing Zar Myint, Zhuoyu Zhou, Qiandai Shi, Junming Chen, Xinyu Dong, Yongmei Xia

**Affiliations:** 1 State Key Laboratory of Food Science and Resource, Jiangnan University, 1800 Lihu Avenue, Wuxi 214122, China; 7170610901@stu.jiangnan.edu.cn (K.Z.M.); 7200610016@stu.jiangnan.edu.cn (Z.Z.); 7220610005@stu.jiangnan.edu.cn (Q.S.); 7150610008@stu.jiangnan.edu.cn (J.C.); 6200612041@stu.jiangnan.edu.cn (X.D.); 2 Key Laboratory of Synthetic and Biological Colloids (Ministry of Education), School of Chemical and Materials Engineering, Jiangnan University, 1800 Lihu Avenue, Wuxi 214122, China

**Keywords:** *stevia*, polyphenol, antimicrobial, anti-inflammatory, digestive enzyme

## Abstract

Polyphenols from stevia leaves (PPSs) are abundant byproducts from steviol glycoside production, which have been often studied as raw extracts from stevia extracts for their bioactivities. Herein, the PPSs rich in isochlorogenic acids were studied for their antimicrobial and anti-inflammatory properties, as well as their inhibitory effects on digestive enzymes. The PPSs presented stronger antibacterial activity against *E. coli*, *S. aureus*, *P. aeruginosa*, and *B. subtilis* than their antifungal activity against *M. furfur* and *A. niger*. Meanwhile, the PPSs inhibited four cancer cells by more than 60% based on their viability, in a dose-dependent manner. The PPSs presented similar IC_50_ values on the inhibition of digestive enzyme activities compared to epigallocatechin gallate (EGCG), but had weaker anti-inflammatory activity. Therefore, PPSs could be a potential natural alternative to antimicrobial agents. This is the first report on the bioactivity of polyphenols from stevia rebaudiana (*Bertoni*) leaves excluding flavonoids, and will be of benefit for understanding the role of PPSs and their application.

## 1. Introduction

Stevia leaf extract contains a variety of bioactive ingredients such as phenols, flavonoids, sterols, terpenes, tannins, vitamins, and minerals [1,2,3,4], and many of these may be responsible for antioxidant, antimicrobial, anti-inflammatory, anti-obesity, and anti-cancer activities [5,6,7]. Among them, polyphenols from *Stevia* (PPS) represented about 2–4% of the dried Stevia leaves. They comprise mainly various chlorogenic acids or their analogues, which makes PPSs an abundant and renewable biomaterial as a potential phenolic antioxidant and antimicrobial agent with anti-inflammatory properties [8,9].

However, most reports are based on plant extracts containing mixtures of polyphenols and flavonoids that all have bioactive effects [5,10]. For example, the stevia extract inhibited the DPPH radical scavenging activity, ranging from 71.92% to 87. 96%, which was produced by 113.8 mg/g dw of phenols together with 49.13 mg/g of flavonoids [11].

Moreover, it is very difficult to cross-check and compare the results from the literature on the antibacterial activity of the stevia phenolic extracts, even if not comparable using orders of magnitude of the MIC values towards the same bacterial strain. For instance, the antibacterial potential of *Stevia* crude extract against four bacteria (*E. coli*, *S. aureus*, *P. aeruginosa*, *C. albicans*) was found to be 1.25–5 mg/mL for the minimum inhibitory concentration (MIC) [12], in which the methanol extract contained phenols and flavonoids of 99.19–129.01 mg gallic acid equivalents/g, and 73.9–113.4 mg quercetin equivalents/g, respectively. Solvent (hexane, methanol, ethanol, ethyl acetate, and chloroform) extracts from stevia leaves exhibited MIC values (mg/mL) of 30, 120, 120, 60, and 60, with inhibition zones ranging from 9 mm to 17.3 mm for the MIC against 16 bacterial strains of the genera *Streptococcus* [13]. Another report presented very low MICs at µg/mL levels [14], that is, solvent (petroleum ether, alcohols, ethyl acetate, chloroform) extracts from stevia flowers or leaves resulted in 0.390 to 25 µg/mL of MIC against *B. subtilis*, *K. pneumonia*, *P. vulgaris*, *S. pneumoniae*, *S. aureus,* and *P. fluorescence*. The confusion may mainly be due to the different phenol content and the composition of the samples of stevia phenol extracts. The worst aspect for cross-checking is that the experiments were lacking positive controls in the MIC test, although they did set controls in the inhibition zone test. It can be seen from the above literature, except for reference [14], that the organic solvent polarity did affect the MIC but did not generate differences in orders of magnitude in the MIC values for the phenolics from stevia [12,13].

In addition to the antimicrobial potential, the ethanolic extract of the leaves presented anti-inflammatory activity, depending on the different leaf drying methods used [15], which might be due to the influence of drying methods on total phenolics content (TPC).

Therefore, the bioactivities of the PPSs have not been adequately investigated. This study aims to evaluate the antimicrobial and cytotoxic activity of PPSs that exclude flavonoids, and their capacity to inhibit digestive enzymes and their anti-inflammatory activity in comparison to the popular polyphenol, EGCG. This information will be beneficial to the development of PPSs as natural healthcare or a personal care material.

## 2. Results and Discussion

### 2.1. Cytotoxicity of PPSs towards Cancer Cells

The extracts from the *Stevia rebaudiana* leaves were reported to be non-cytotoxic against healthy cell lines and macrophage RAW 264.7 cells at concentrations up to 5 µg/mL, but showed cytotoxicity against HeLa cancer cells at an IC_50_ of 50 µg/mL, in which the total phenols and flavonoids were 80.13–86.47 mg gallic acid/g extract, and 111.16–126.70 mg quercetin/g extract, respectively [16]. Additionally, Stevia extract obtained at the flowering phase could reduce cell viability to 55% against MDA MB231 at 1 mg/mL, while extracts from other parts displayed a cell viability below 70%. Some other plant extracts containing both polyphenols and flavonoids were also reported to have cytotoxicity on cancer cells. For example, the methanolic extract of *T. articulata* (409.92 ± 6.03 mg GAE/g extract of phenols, 177.71 mg QE/g extract of flavonoids) displayed antiproliferative activity against MCF-7 and PKO cells with IC_50_ values in the range of 219–325 µg/mL [17].

**Figure 1:**
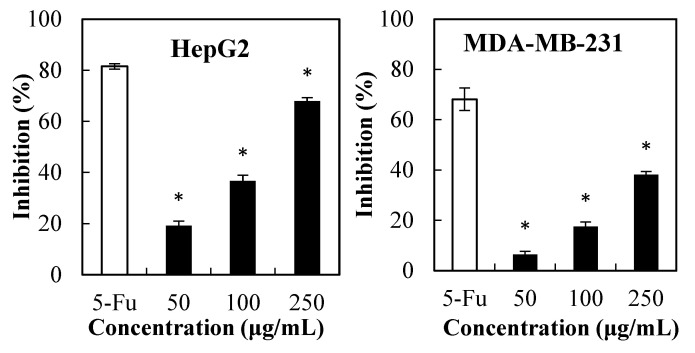

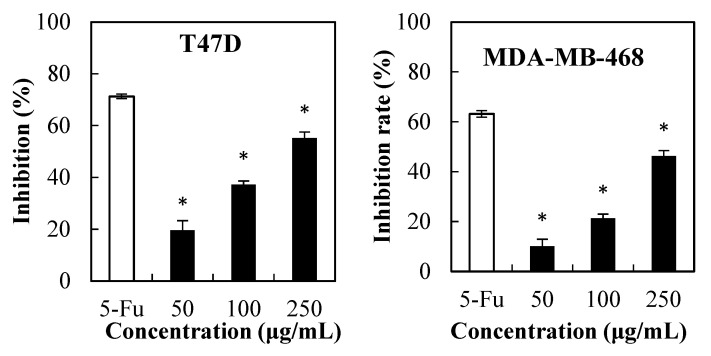
MTT assay of the PPS cytotoxicity on the cancer cell lines of HepG2, MDA-MB-231, T47D, and MDA-MB-468. Data are presented as mean ± SD, *n* = 3, * *p* < 0.05 compared with the value of 5-fluorouracil (5-FU, positive control).

Herein, the MTT test was conducted to determine the effect of PPSs (50 µg/mL–250 µg/mL) against liver hepatocellular carcinoma cells and three breast cancer cells (Figure 1), where the cytotoxic effect of PPSs on the tested cells was dose-dependent. The IC_50_ of the PPSs was 170.54, 322.58, 269.42, and 207. 75 µg/mL for HepG2, MDA-MB-231, MDA-MB-468, and T47D cells, respectively.

Previous reports found that ethanolic and water extracts from Stevia leaves presented no cytotoxicity towards HepG2 cells [18] and healthy cells [10], which could be due to insufficient PPSs content. Another possible reason for PPSs (Figure 1) showing higher cytotoxicity towards HepG2 cells could be that the PPSs used in this experiment possessed higher total phenolics content (771 mg GAE/g extract) and were rich in isochlorogenic acids (98.7% in the total polyphenols (Figure S1, Table S1, Supplementary Materials).

### 2.2. Antimicrobial Activity

Plant polyphenols have been applied as a strategy of defense against phytophagous insects, fungi, or bacteria [1]. Herein, antimicrobial activity was assayed using both the inhibition zone assay and MIC assay. As aforementioned, stevia extracts have been found to possess antimicrobial activity. There is also no detailed study regarding the antimicrobial activity of PPSs excluding other phytochemicals from stevia extracts. Solvent extracts from stevia presented inhibition zones of 11–13 mm at best, compared to 8–14 mm yielded by tetracycline, against *B. subtilis*, *K. pneumonia*, *P. vulgaris*, *S. pneumoniae*, *S. aureus*, and *P. fluorescence* [14]; unfortunately, the authors only reported on the qualitative analysis of the extracts, so the comparison actually only worked between the extracts.

**Figure 2:**
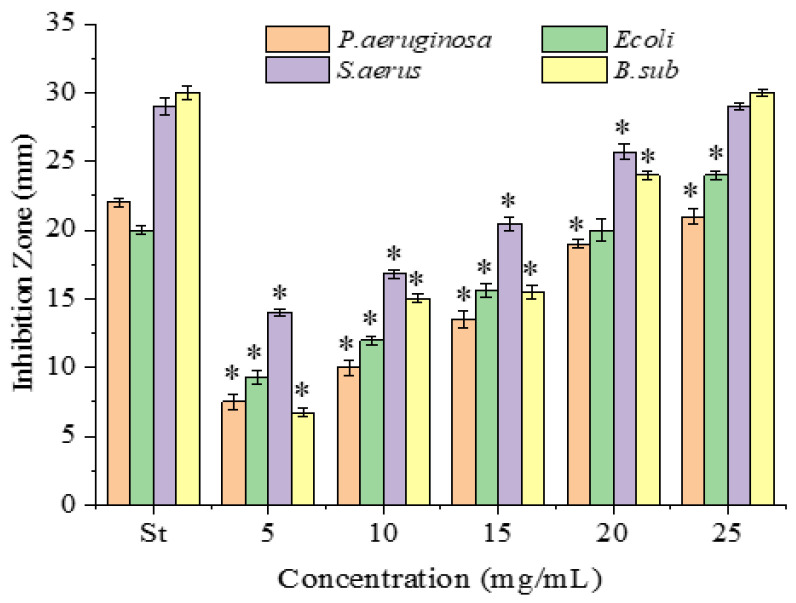
Antibacterial activity of PPSs against *P. aeruginosa* (KCTC 2651), *E. coli* (ATCC 25922), *B. subtilis* (ATCC 6633), and *S. aureus* (ATCC 25923). St represents tetracycline hydrochloride, 0.1 mg/mL. Data are presented as mean ± SD, *n* = 3, * *p* < 0.05 compared with the value for tetracycline hydrochloride.

**Figure 3:**
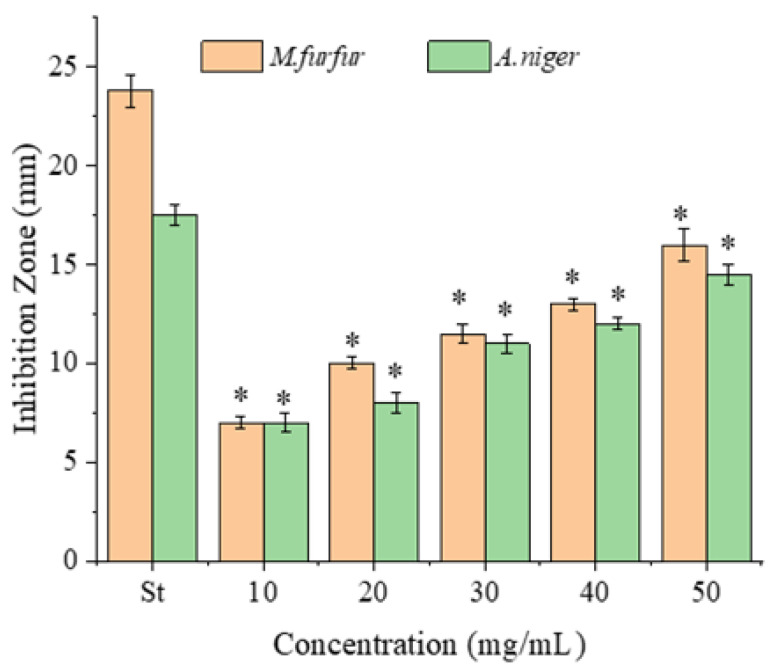
Antifungal activity of the PPS against *M. furfur* and *A. niger.* Data are presented as mean ± SD, *n* = 3, * *p* < 0.05 compared with the value of the positive control (St, Fluconazole, 1 mg/mL).

The antimicrobial activities of the PPSs were examined against four bacterial (Figure 2) and two fungal strains, with tetracycline hydrochloride as the positive control (Figure 3).

All inhibitions were concentration-dependent. Obviously, compared to the positive control, tetracycline hydrochloride (Figure 2), the PPSs presented much weaker antimicrobial activity based on the dosage applied. However, the PPSs presented a higher activity against Gram-positive than Gram-negative bacteria, which agreed with reports using other bioactive components (polyphenols, tannins, alkaloids, and flavonoids) [19,20,21]. This might be due to the lipopolysaccharide on the surfaces of the Gram-negative bacteria [21]. Sadly, considering the PPSs dosage applied in the experiment, the antibacterial activity of the PPSs was much weaker than that of tetracycline hydrochloride.

As for fungi, *A. niger* is a typical fungus affecting food and human health, whereas *M. furfur* is used for dandruff issues. The two fungi both have fluconazole as the effective inhibitor [22]. The antifungal activity of the PPSs against *A. niger* and *M. furfur* (ATCC 44344) was assayed using fluconazole as the positive control (Figure 3). The two fungal strains were found to be similarly sensitive to the PPSs, with inhibition zones of 7–16 mm for *M. furfur* (ATCC 44344) and 7–14.5 mm for *A. niger.*

In general, polar solvent extracts have higher antimicrobial activities than non-polar solvents [23,24]. The PPSs assayed in this experiment were extracted using water. The MIC value of the PPSs against fungi after 48 h of incubation was higher than for bacteria.

For longer-term inhibition, that is, the MIC/MBC value, the antibacterial activity against all tested bacteria was in the range of 1.67–3.33 mg/mL (Table 1).

Hence, the most susceptible strain was Gram-positive bacteria (MIC or MBC = 1.67–3.33 mg/mL). This finding is similar to the literature showing that the PPSs [25], or other polyphenols had lower antibacterial activity on Gram-negative bacteria, due to their cell permeability [21].

In summary, considering the dosage, PPSs could be applied as a natural preservative to prevent food poisoning and preserve food, and to alternate synthetic antimicrobial agents, since the assayed bacterial and *A. niger* strains are important in food spoilage and poisoning.

### 2.3. Inhibitory Effect of PPSs on Digestive Enzymes

The inhibitory effect of polyphenols on digestive enzymes is one of the mechanisms that allow polyphenols to provide anti-obesity activity. Different inhibition activities against digestive enzymes were observed in polyphenols from various extracts. For instance, grape seed extract was found to strongly inhibit α-amylase and α-glucosidase [26]. All fractions of *Syzygium cumini* extract were capable of inhibiting α-amylase by 100%, and the fractions with DCM, EtOAc, and ButOH inhibited α-glucosidase more than 50% [27].

**Figure 4:**
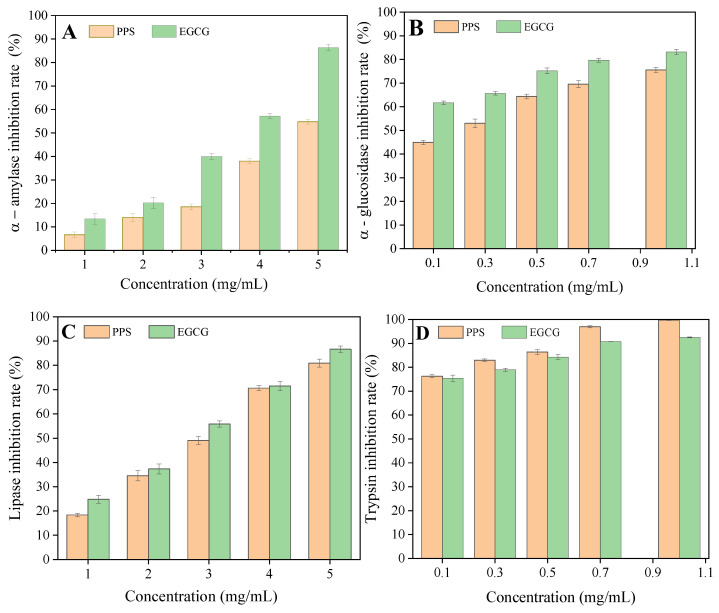
Inhibition rates for α-amylase (**A**), α-glucosidase (**B**), lipase (**C**), and trypsin (**D**) by the PPSs. Values are expressed as means ± SD (*n* = 3). There was no significant difference between the data from EGCG and PPS.

In this study, the PPSs were found to exhibit a similar inhibitory effect against digestive enzymes when compared to EGCG (Figure 4, Table 2). The PPSs inhibited both carbohydrate hydrolyzing enzymes in a dose-dependent manner. It is interesting that α-glucosidase was more sensitive than the α-amylase; the IC_50_ values were 0.19 mg/mL and 4.97 mg/mL for α-glucosidase and α-amylase, respectively. Our result is consistent with that reported using *Ajugaiva Schreber* extracts, in which the IC_50_ against α-glucosidase was lower than that of α-amylase [28], but is opposite to that reported by Franco et al. [27].

The PPSs assayed were rich in isochlorogenic acids, which possess more phenolic hydroxyl groups (four) than chlorogenic acid (two phenolic hydroxyl groups in the molecule), but less than that in EGCG (eight phenolic hydroxyl groups in the molecule). This may be one of the reasons why EGCG exhibited slightly stronger inhibition.

Nevertheless, the inhibitory effect of the PPSs on digestive enzymes had a similar IC_50_ to EGCG. Hence, the PPS may act to potentially inhibit the activities of digestive enzymes.

### 2.4. Kinetic Analysis of the Inhibition of Lipase and Trypsin

**Figure 5:**
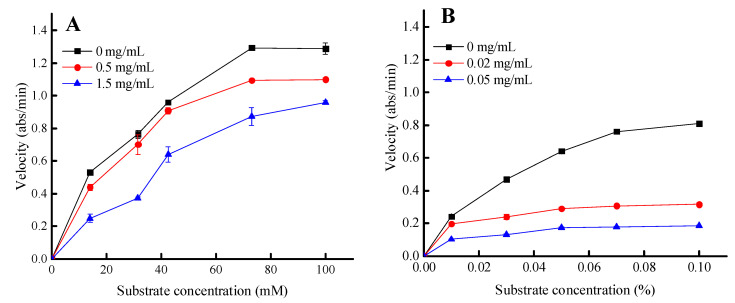
The inhibitory kinetics of the PPSs on lipase (**A**) and trypsin (**B**) were determined using Michaelis–Menten analysis. Values are expressed as the mean ± SD (*n* = 3).

Trypsin and lipase were selected for further kinetic analysis to investigate the inhibitory mechanism, since these two enzymes were slightly more sensitive to the PPSs compared to EGCG. The initial velocity of hydrolysis catalyzed by trypsin and pancreatic lipase was determined at various substrate concentrations in the presence and absence of the PPSs (Figure 5).

The inhibitory kinetics of the PPSs on the trypsin-catalyzed BAPNA substrate were reduced for both Vmax and Km values. These results suggest a competitive inhibition mechanism (Figure 5, Table 3).

### 2.5. In Vitro Anti-Inflammatory Activity

The anti-inflammatory activity of isochlorogenic acids, such as 4,5-dicaffeoylquinic acid or 5-O-caffeoylquinic acid, was found using RAW264.7 cells and a rat model [29], or endogenous antioxidant enzymes [30]. However, protein denaturation and excessive uric acid have been associated with the inflammatory response. This can be caused by xanthine oxidase which catalyzes the oxidation of hypoxanthine to xanthine and then to uric acid [31].

**Figure 6:**
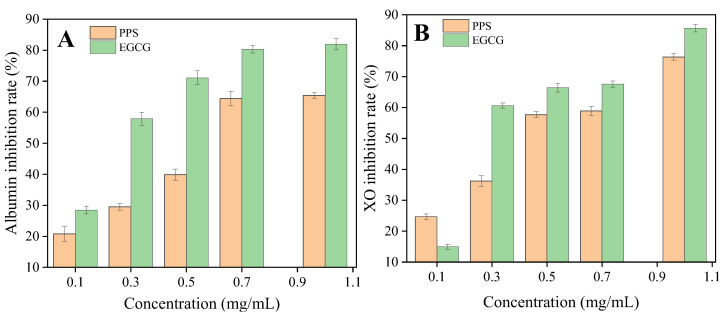
Effect of the PPSs on (**A**) albumin inhibition rate and (**B**) XO inhibition rate. Values are expressed as mean ± SD (*n* = 3).

The inhibition of XO and albumin denaturation by the PPSs are presented in Figure 6, with EGCG as a reference. The IC_50_ of the PPSs for each activity was higher than that of EGCG. A different study [15] found that the Stevia extract exhibited a weaker anti-inflammatory effect than the controls, nimesulide and indomethacin. Nevertheless, the results contribute evidence for the anti-inflammatory activity of the PPS.

## 3. Materials and Methods

### 3.1. Materials

PPSs (Batch number 20181214, 771 mg GAE/g extract, 54.5% of weight content of total polyphenols) extracted with water from dried stevia leaves were gifts from Zhucheng HaoTian Pharm Co., Ltd., Shandong, China. 3-[4,5-dimethyl-2-thiazolyl)-2,5-diphenyl-2H-tetrazolium bromide (MTT), trypsin-EDTA solution, penicillin, and streptomycin were purchased from Beyotime Biotechnology Co., Ltd. (Shanghai, China). 4(2-hydroxyethyl)-1-piperazineethane sulfonic acid (HEPES), DMEM, and fetal bovine serum were purchased from Gibco Life Technologies Corporation (Carlsbad, CA, USA). Nine phenolic standards (chlorogenic acid, isochlorogenic acid A, B, C, caffeic acid, quercetin, galuteolin, crytochlorogenic acid, and neochlorogenic acid), porcine pancreas α-amylase (5 U/mg solid, Type VI-B, amylase activity), α-glucosidase (10 U/mg protein, glucosidase activity) from *Saccharomyces cerevisiae*, trypsin from porcine pancreas (1000 U/mg solid), porcine pancreas lipase (100 U/mg protein, Type II, lipase activity), and xanthine oxidase from bovine milk (0.4 U/mg protein) were purchased from Sigma Aldrich, USA. Benzoyl-DL arginine-*p*-nitroanilide (BAPNA, ≥99% TLC purity), *p*-nitrophenyl butyrate (pNPB), and *p*-nitrophenol-α-d-Glucopyranoside (pNPG), xanthine, tetracycline hydrochloride, and fluconazole were purchased from Aladdin, Shanghai Cooperation, China. 3,5-dinitro salicylic acid was purchased from Shanghai Macklin Biochemical Co., Ltd. Disodium hydrogen phosphate dodecahydrate, sodium dihydrogen phosphate dihydrate, sodium carbonate, nutrient agar, potato dextrose agar, egg albumin, dimethyl sulfoxide (DMSO), and 5-fluorouracil (5FU) were purchased from Sinopharm Chemical Reagent Co., Ltd., Shanghai, China. All the chemicals were of analytical grade and used as received unless stated specifically.

### 3.2. HPLC Analysis of the PPS

The analysis was performed using an Agilent 1260 series HPLC instrument equipped with an Agilent Zorbax Extend-C18 (4.6 × 250 mm, 5 µm) and a DAD detector (Thermo Fisher Scientific, Waltham, MA, USA). The flow rate of mobile was 1.0 mL/min, running as follows: 0–5 min (95% A–5% B), 5–30 min (83% A–17% B), 30–50 min (78% A–22% B), and 50–55 min (78% A–22% B). Mobile phase: A was 0.2% phosphoric acid, while B consisted of acetonitrile. Quantification was carried out at 330 nm. The polyphenol content was expressed in wt%. A set of calibrations of concentration peak areas was made with standard samples and used for calculating the concentration of the polyphenols. The HPLC profile of the PPSs is presented in Figure S1 (Supplementary Materials).

### 3.3. Microbial Strains, Cancer Cell Lines, and Cell Culture

*Pseudomonas aeruginosa* (KCTC 2651) was from Biovector Science Lab, Inc, Beijing, China; *E. coli* (ATCC 25922), and *B. subtilis* (ATCC 6633), *S. aureus* (ATCC 25923), *A. niger* (CICC 2487), and *Malassezia furfur* (ATCC 44344) were purchased from Guangdong microbial culture collection center, Guangdong, China.

The human liver hepatocellular carcinoma epithelial cell HepG2 (CC0119), two human breast adenocarcinoma epithelial cells MDA-MB-231 (CC0301), MDA-MB-468 (CC0309), and human breast carcinoma epithelial cell T47D (CC0303) were supplied from Guangzhou Cellcook Biotech Co., Ltd., Guangzhou, China. All of the cell lines were confirmed using STR. The cell lines were grown in DMEM with high glucose (GE Healthcare, USA) supplemented with 10% fetal bovine serum, penicillin (100 U/mL), streptomycin (100 μg/mL), and 10 mM 4(2-hydroxyethyl)-1-piperazineethanesulfonic acid (HEPES). The cells were then used for the following analyses when they reached the logarithmic growth phase.

### 3.4. Cytotoxicity Assay

The effect of the PPSs as a potential anticancer treatment was evaluated using an MTT assay [32]. The cells in the logarithmic growth phase were digested with 0.25% trypsin and adjusted to 5000 cells/well using DMEM complete medium, respectively. Before the PPSs treatment, 100 μL of the cell suspension was pipetted into each well of 96-well plates and cultured for 24 h at 37 °C in 5% CO_2_. Subsequently, the cells were incubated with the PPSs at 37 °C in 5% CO_2_ for up to 48 h. The culture medium was then removed and 100 μL of MTT reagent (0.5 mg/mL in culture medium) was added. After another 4 h of incubation, the MTT/medium was removed and 150 μL of DMSO was added to dissolve the formazan crystals. The absorbance of the solution was recorded at 570 nm (Universal Microplate Reader ELX800NB (Bio-Tek Instruments INC, Vinooski, VT, USA) to calculate the inhibition rate on cell growth. 5-fluorouracil (5FU) was employed as a positive control. All measurements were performed in triplicate. The inhibition rate was calculated as follows:

(1) $$\mathrm{Inhibition}\;\mathrm{rate}\;(\%)=\frac{A_{570}\;\mathrm{of}\;\mathrm{control}-A_{570}\;\mathrm{of}\;\mathrm{sample}}{A_{570}\;\mathrm{of}\;\mathrm{control}}\times 100$$

### 3.5. Antimicrobial Activity

The nutrient media were inoculated with a swab dipped into a bacterial suspension with a turbidity of 10^4^ CFU/mL. The antimicrobial assay was performed using the agar diffusion method, according to the literature [33,34], with some modifications. In short, 0.1 mL of the PPSs solution was loaded onto sterile blank discs (6 mm in diameter) and the discs were impregnated onto inoculated agar. Discs with antibiotics (tetracycline hydrochloride for bacteria, and fluconazole for fungi) were used as positive controls, and discs impregnated with solvents were used as negative controls. The culture dish was then placed in an incubator at 37 °C for 24 h with bacteria and at 28 °C for 48 h with fungi. The diameter (mm) of the inhibition zone was measured to indicate antimicrobial activity. The experiment was repeated three times.

### 3.6. Determination of Minimum Inhibitory Concentration (MIC)

The minimum inhibitory concentration (MIC, mg/mL), the minimum bactericidal concentration (MBC, mg/mL), and the minimum fungicidal concentration (MFC, mg/mL) for the PPSs were determined according to the literature [35]. Briefly, the prepared broth solution (14 mL) was added to each PPSs dilution (1 mL) in the Petri dish under sterile conditions. Subsequently, 0.1 mL of the prepared bacterial or fungal suspensions were poured onto the culture medium and incubated for 24 h at 37 °C. The lowest concentration of the PPSs at which there was no strain growth was recorded as the minimum inhibitory concentration (MIC). To measure the MBC and MFC, the plates without strain growth were incubated again for another 24 h and 48 h, and the lowest concentration at which no growth occurred was considered as the MBC and MFC.

### 3.7. Inhibitory effect of the PPSs on Digestive Enzymes

#### 3.7.1. Pancreatic Lipase Assay

Lipase inhibition activity was determined according to the literature [36,37], with minor modifications. Briefly, 100 µL of the prepared PPL solution was mixed with either 200 µL of the PPSs, or control solution in a tube. The mixture was amended to 1 mL withthe phosphate buffer and preincubated for 5 min at 37 °C. Then, 100 µL of P-nitrophenyl butyrate (PNPB) was added into the above tube and incubated again for 10 min at 37 °C. Lipase activity was calculated by measuring the hydrolysis of the PNPB to p-nitrophenol at 410 nm.

#### 3.7.2. α-Amylase Assay

The α-amylase inhibition assay was assessed according to the literature [38]. A total of 0.5 mL of sample solution was added to each tube, including 0.5 mL of PPA solution (0.5 mg/mL), and incubated at 37 °C for 10 min. Then, 0.5 mL of a 1% starch solution was added to the above test tube and kept at 37 °C for another 30 min and the reaction was terminated with DNS reagent (0.5 mL). The test tubes were then kept in a boiling water bath for 5 min, cooled to room temperature, and diluted with water (final volume of 10 mL). The absorbance of the reaction mixture was recorded at 540 nm. The control sample was prepared as above with phosphate buffer instead of sample solution. The percent inhibition rate of α-amylase was calculated as follows:

(2) $$Inhibition\;rate\;(\%)=\frac{A_{540}\;\mathrm{of}\;\mathrm{control}\;-\;A_{540}\;\mathrm{of}\;\mathrm{sample}\;}{A_{540}\;\mathrm{of}\;\mathrm{control}}\times 100$$

#### 3.7.3. α-Glucosidase Assay

α-Glucosidase inhibition assay was carried out using the method from Ghane et al. [39]. The reaction mixture consisted of sample solution (20 µL), 0.1 mL α-glucosidase (1 U/mL), and 0.5 mL phosphate buffer (0.1 M, pH 6.9). After incubating the mixture for 5 min at 25 °C, 0.1 mL of p-nitrophenol-α-d-glucopyranoside (pNPG, 5 mM, in PBS buffer) was added as the substrate into the above mixture to initiate the reaction. The mixture was incubated again at 25 °C for 10 min and then terminated using a sodium carbonate solution (1 mL, 0.1 M). The solution absorbance was recorded at 405 nm. The control was made with 20 µL of phosphate buffer (0.1 M, pH6.9) without sample. The inhibition rate of the PPSs to α-glucosidase was calculated in the same way as that for α-amylase (Equation (2)).

#### 3.7.4. Trypsin Assay

The inhibitory effect of the PPS against the trypsin enzyme was assessed using benzoyl-DL arginine-p-nitroanilide (BAPNA) as the substrate. A total of 1 mL enzyme solution and 0.5 mL sample solution were preincubated at 37 °C for 10 min. Then, 2.5 mL of BAPNA solution (0.04%) in Tris-HCl buffer (50 mM, pH 8.2, containing 20 mM CaC1_2_) was added and the mixture was incubated in a boiling water bath at the same temperature and for the same length of time. Next, 0.5 mL of acetic acid solution (30% *v*/*v*) was poured into the above mixture to terminate the reaction. The solutions were filtered and the absorbance was read at 410 nm [40]. The inhibition rate (%) was calculated using the following equation:

(3) $$Inhibition\;rate\;(\%)=\frac{A_{410}\;\mathrm{of}\;\mathrm{trypsin}\;-\;A_{410}\;\mathrm{of}\;\mathrm{sample}\;}{A_{410}\;\mathrm{of}\;\mathrm{trypsin}\;}\times 100$$

### 3.8. Kinetics Analysis

The inhibition assays for lipase and trypsin were performed using the analysis of Michaelis–Menten kinetics, with PNPB and BAPNA as substrate and with different concentrations of the PPSs, respectively. The sample absorbance was recorded at 410 nm. The maximum velocity (Vmax), Michaelis–Menten constant (Km), and the mode of inhibition were determined from the curve of the PPS concentration vs. the enzymatic activity [41].

### 3.9. In Vitro Anti-Inflammation

#### 3.9.1. Inhibition of Albumin

Egg albumin solution (5%, 0.2 mL), 2.8 mL of phosphate buffered saline (PBS, pH 6.8), and 2 mL of different concentrations of sample solution (0.1–1 mg/mL) were incubated at 37 °C for 15 min [42]. The temperature was then raised to 70 °C for 5 min. After cooling the mixture, the absorbance was measured at 660 nm and distilled water served as the control. The inhibition rate of albumin was calculated using the following formula:

(4) $$Inhibition\;rate\;(\%)=\frac{A_{660}\;\mathrm{of}\;\mathrm{sample}\;-\;A_{660}\;\mathrm{of}\;\mathrm{control}}{A_{660}\;\mathrm{of}\;\mathrm{control}}\times 100$$

#### 3.9.2. Inhibition of Xanthine Oxidase

The inhibition of xanthine oxidase (XO) was measured using xanthine as the substrate using a modified method [43]. The reaction mixture comprised 2.7 mL of buffer solution (phosphate buffer, 0.2 M, pH 7.5), 100 µL of 10 mM xanthine solution, and 20 µL of the test sample, all in phosphate buffer solution (0.2 M, pH 7.5). Xanthine oxidase (200 µL, 0.2 U/mL) was added to the above mixture and was then incubated for 30 min at 37 °C. The reaction was terminated with 0.5 M HCl (500 µL). The sample absorbance was recorded at 290 nm against the control solution prepared the same way without enzyme. The XO enzyme inhibition rate was calculated as follows:

(5) $$Inhibition\;rate\;(\%)=(1-\frac{A_{290}\;\mathrm{of}\;\mathrm{sample}}{A_{290}\;\mathrm{of}\;\mathrm{control}})\times 100$$

### 3.10. Statistical Analysis

The assay was conducted using three replicates for each treatment. Data were expressed as mean ± SD values. Statistical differences were determined using Student’s *t*-test. *p* < 0.05 was considered to be statistically significant.

## 4. Conclusions

Polyphenols from plant extracts are gaining interest for new applications, which allows the quick screening of the biological properties of the PPSs via in vitro assays. Depending on the assayed food or dandruff-related microbial, PPSs presented higher inhibition against bacteria than fungi, with a MIC range of 1.67–3.33 mg/mL for bacteria and 6.67–13.3 mg/mL for fungi. Meanwhile, the PPS exhibited strong inhibitory effect on the digestive enzymes with similar IC_50_ values to EGCG.

Therefore, the PPSs could be a potential ingredient for cosmetics and food. However, future research will be needed to elucidate the anti-inflammatory mechanisms in vivo, as well as the bioavailability and metabolic pathways. In addition, although aqueous phenolic extracts possess high solubility in water, the extracts with organic solvents may provide different or even better bioactivity. 

## Figures and Tables

**Table 1:** MIC, MBC, and MFC values for the PPSs against testing microorganisms.

	PPS		Positive Control *	
	MIC (mg/mL)	MBC/MFC (mg/mL)	MIC (µg/mL)	MBC/MFC (µg/mL)
*B. subtilis*	1.67	3.33	4	8
*S. aureus*	1.67	3.33	4	8
*P. aeruginosa*	3.33	≥3.33	1.6	3.2
*E. coli*	3.33	≥3.33	1.6	3.2
*M. furfur*	6.67	13.33	33	67
*A. niger*	6.67	13.33	33	67

* Tetracycline hydrochloride for bacteria and fluconazole for *fungi.*

**Table 2:** IC_50_ values (mg/mL) for the PPS inhibition of typical digestive enzymes.

Sample	α-Amylase	α-Glucosidase	Lipase	Trypsin
PPS	4.96 ± 0.09	0.19 ± 0.04	3.25 ± 0.06	0.065 ± 0.001
EGCG	4.26 ± 0.07	0.07 ± 0.00	3.72 ± 0.14	0.066 ± 0.001

**Table 3:** Effect of the PPS (mg/mL) on the Vmax and Km values of trypsin and lipase.

Parameters		Lipase			Trypsin	
	0	0.5	1.5	0	0.02	0.05
Vmax	1.67	1.59	1.68	1.07	0.32	0.19
Km	30.77	36.8	83.9	0.03	0.007	0.009
Inhibition type	competitive			uncompetitive		

## Data Availability

No new data was created.

## Supplementary Materials

The following supporting information can be downloaded at: https://www.mdpi.com/article/10.3390/molecules28227572/s1, Figure S1: Vendor provided HPLC profile of the PPS (A), Isochlorogenic acid A (B) and calibration of Isochlorogenic acid A (C). Isochlorogenic acid A was used as the standard to determine the content of other polyphenols. The conversion factors (f) of HPLC peak area (Ai) of each polyphenol over that of isochlorogenic acid A (A0) are as follows (f = Ai/A0): Neochlorogenic acid: 0.69; Chlorogenic acid: 0.69; Cryptochlorogenic acid: 0.69; Isochlorogenic acid B: 1.0; Quercitrin: 0.87; Isochlorogenic acid C: 1.0; Table S1: The composition of the PPS sample from Stevia rebaudiana Bertoni leaves; Figure S2: Antibacterial activity of the PPS against *P. aeruginosa*, *E. coli*, *B. subtilis* and *S. aureus*; Figure S3: Antifungal activity of the PPS against *M. furfur* and *A. niger*.
