# Supplementary material for: Stevia Polyphenols, Their Antimicrobial and Anti-Inflammatory Properties, and Inhibitory Effect on Digestive Enzymes
Source: Molecules. 2023 Nov 13;28(22):7572. doi: 10.3390/molecules28227572 (PMC10673113; doi:10.3390/molecules28227572)
Supplement: Supplementary file 1 [file molecules-28-07572-s001.zip › molecules-2693218-supplementary.pdf]

Supporting Material

Stevia Polyphenols, Their Antimicrobial and Anti-Inflammatory Properties, and Inhibitory Effect on Digestive Enzymes

Khaing Zar Myint 1,2, Zhuoyu Zhou 1,2, Qiandai Shi 1,2, Junming Chen 1, Xinyu Dong 1 and Yongmei Xia 1,2,*

1 State Key Laboratory of Food Science and Resource, Jiangnan University, 1800 Lihu Avenue,
Wuxi 214122, China; 7170610901@stu.jiangnan.edu.cn (K.Z.M.); 7200610016@stu.jiangnan.edu.cn (Z.Z.);
7220610005@stu.jiangnan.edu.cn (Q.S.); 7150610008@stu.jiangnan.edu.cn (J.C.); 6200612041@stu.jiangnan.edu.cn (X.D.)
2 Key Laboratory of Synthetic and Biological Colloids (Ministry of Education), School of Chemical
and Materials Engineering, Jiangnan University, 1800 Lihu Avenue, Wuxi 214122, China
* Correspondence: ymxia@jiangnan.edu.cn

The Main components and polyphenols in the PPS

There are more than 39 phenolic compounds in *stevia rebaudiana* (bertoni) have been disclosed. In this experiment, seven phenolic compounds (chlorogenic acid, isochlorogenic acid A, B, C, cryptochlorogenic acid, neochlorogenic acid, and quercetin) were observed in the assayed PPS as main components (Table S1, Figure S1).

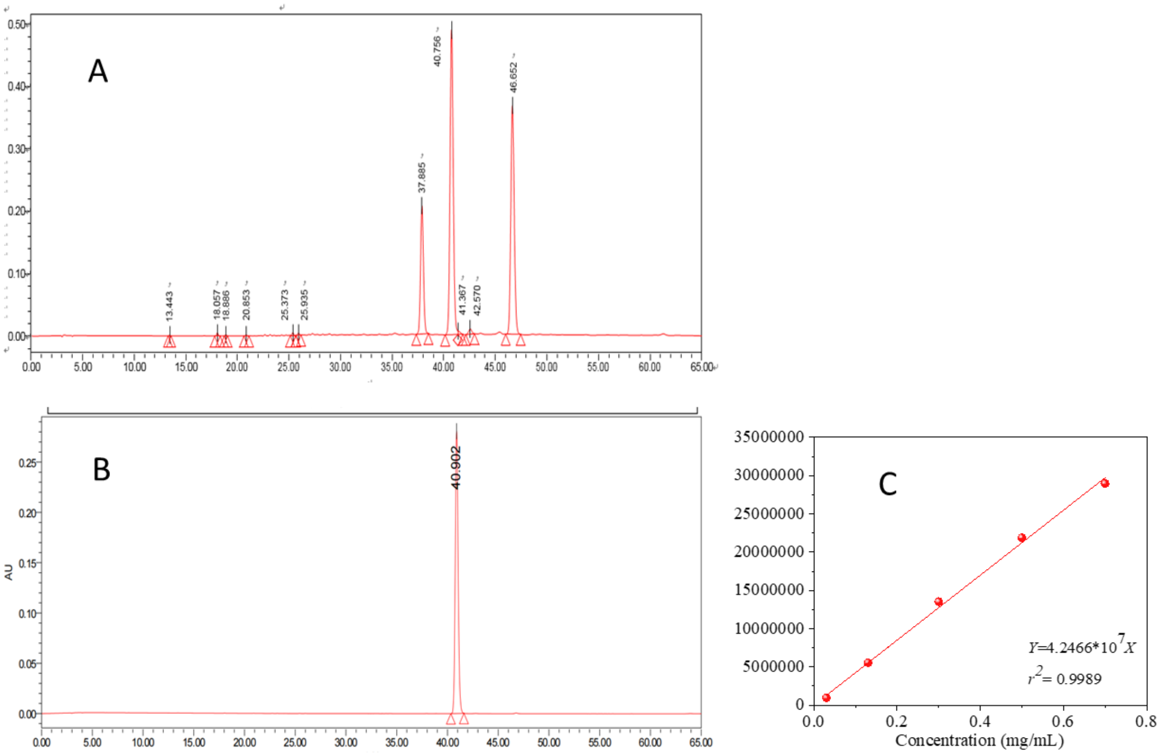

Figure S1. Vendor provided HPLC profile of the PPS (A), Isochlorogenic acid A (B) and calibration of Isochlorogenic acid A (C). Isochlorogenic acid A was used as the standard to determine the content of other polyphenols. The conversion factors (f) of HPLC peak area (Ai) of each polyphenol over that of isochlorogenic acid A (A0) are as follows (f= Ai/ A0): Neochlorogenic acid: 0.69; Chlorogenic acid: 0.69; Cryptochlorogenic acid: 0.69; Isochlorogenic acid B: 1.0; Quercitrin: 0.87; Isochlorogenic acid C: 1.0.

Table S1. The composition of the PPS sample from *Stevia rebaudiana* Bertoni leaves

Composition	Contents (wt.%, HPLC)[1]	Retention time (min)
Neochlorogenic acid	0.1	13.443
Chlorogenic acid	0.48	18.057
Cryptochlorogenic acid	0.10	18.886
Caffeic acid	0	-
Galuteolin	0	-
Isochlorogenic acid A	26.18	40.756
Isochlorogenic acid B	6.59	37.885
Isochlorogenic acid C	21.26	46.652
Quercetin	0.01	41.367
Protein	0.82	
Total flavonoid	0.44	
Polysaccharide	31.64	
Water	4.5	

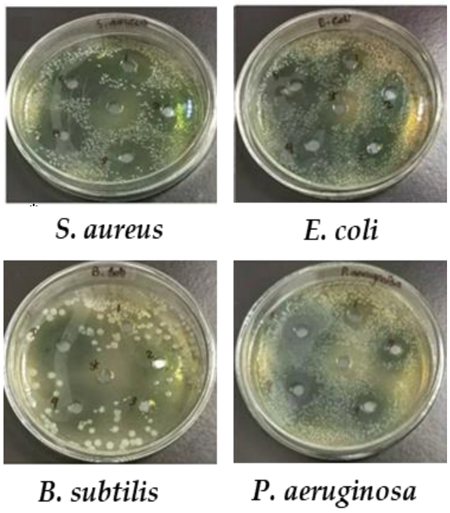

Figure S2. Antibacterial activity of the PPS against *P. aeruginosa, E. coli, B. subtilis* and *S. aureus.*

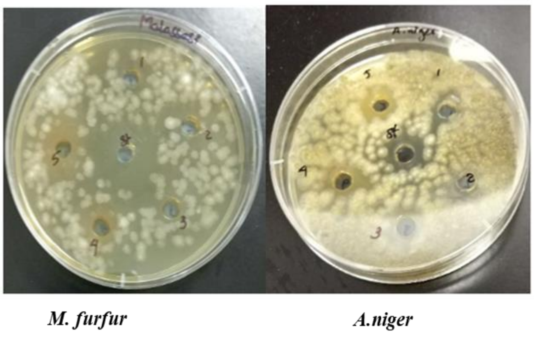

Figure S3. Antifungal activity of the PPS against *M. furfur* and *A.niger.*

1. Myint, K. z.; Zhou, Z.; Xia, Y.; Fang, Y.; Wu, M.; Zhu, S.; Shen, J., Stevia polyphenols: A stable antioxidant that presents a synergistic effect with vitamin C. *J. Food Process. Preserv.* **2021,** 45, (4), e15317.
